# Valgus knee bracing may have no long-term effect on pain improvement and functional activity in patients with knee osteoarthritis: a meta-analysis of randomized trials

**DOI:** 10.1186/s13018-020-01917-x

**Published:** 2020-09-01

**Authors:** Yinuo Fan, Zhongfeng Li, Haitao Zhang, Guoju Hong, Zhongshu Wu, Weifeng Li, Lixin Chen, Yunlong Wu, Qiushi Wei, Wei He, Zhenqiu Chen

**Affiliations:** 1 The First Clinical Medical College, Guangzhou University of Chinese Medicine, 12 Jichang Road, Baiyun District, Guangzhou, 510405 Guangdong Province China; 2 Department of Joint Diseases, The Third Affiliated Hospital of Guangzhou University of Chinese Medicine, NO. 261 Longxi Road, Liwan District, Guangzhou, Guangdong Province China; 3 The Department of Orthopedics, The First Affiliated Hospital of Guangzhou University of Chinese Medicine, Jichang Road 16#, District Baiyun, Guangzhou, 510405 Guangdong Province China

**Keywords:** Knee osteoarthritis, Valgus knee bracing, Pain, Functional activity, Meta-analysis

## Abstract

**Background:**

Knee osteoarthritis (KOA), with a high incidence in old-age population, adversely affects their life quality. The valgus knee bracing is an important physical therapy for KOA, but its clinical effects on pain release and functional improvement remained unclear. This meta-analysis is to systematically evaluate the clinical outcomes of valgus knee bracing in patients with KOA.

**Methods:**

A meta-analysis of clinical randomized controlled trials (RCTs) on pain and functional changes in patients with KOA after using valgus knee braces. The search period was ranged from the inception of the database to May 2020. The enrolled research databases included PubMed, Embase, and Web of Science databases. Two investigators independently formulated inclusion criteria and exclusion criteria and screened and determined the final enrolled literature. Then the outcome indicators were extracted and organized from the included literature, and the risk of bias was assessed by Cochrane Handbook 5.0.1.

**Results:**

A total of 10 articles were included in this study, including 739 patients. Eight articles were related to the visual analog scale (VAS) pain score, and the results showed that RR = − 0.29, 95% CI − 0.73, 0.15], *P* = 0.20; four articles were related to the Western Ontario and McMaster Universities Osteoarthritis Index (WOMAC) function score, and the results showed that RR = − 0.15, 95% CI [− 0.41, 0.11], *P* = 0.26; two articles were related to the Knee Injury and Osteoarthritis Outcome Score (KOOS), and the results showed that RR = 0.58, 95% CI [− 4.25, 5.42], *P* = 0.81; and three articles were related to the KOOS Activities of Daily Living (KOOS-ADL), and the results showed that RR = 0.04, 95% CI [− 0.62, 0.69], *P* = 0.91. These results indicated that the valgus knee bracing has no statistical significance in pain and functional activity improvement of patients with KOA. The subgroup analysis showed that the follow-up time was the source of the heterogeneity of the VAS pain score.

**Conclusion:**

Our current evidence suggests that valgus knee bracing may not improve pain release and function activates in KOA patients in the long-term period, but only being beneficial to the short-term rehabilitation.

## Introduction

Osteoarthritis (OA) is a worldwide degenerative joint disease, being one of the main causes of joint disability [1]. The incidence of osteoarthritis increases with age. As the global population aging, it will have a huge social burden [2]. Knee osteoarthritis (KOA) accounts for 83% of OA [3]. The incidence of KOA is as high as 44% globally, and particularly, the figure for women is much higher than that of men [4, 5]. Early KOA advocates conservative treatment, including oral analgesics, articular injection drugs, physical therapy, and so on [6–8], but these methods did not effectively resolve KOA. In the late stage of KOA, total knee arthroplasty (TKA) is usually the final choice for the KOA patients. However, even though TKA has excellent clinical outcomes, increasing complications such as aseptic loosening of prosthesis, periprosthetic fractures around, and periprosthetic infection are found after surgery. Patients may undergo TKA revision if they suffered from these disastrous complications [9]. Therefore, it is greatly urgent to develop conservative therapies for effective treatment of KOA, in order to reach high daily-life quality or delay the time of TKA treatment.

Valgus knee bracing is found to effectively correct the lower limb force lines, release pain feelings, and improve joint functions of KOA patients by optimizing their biomechanical axis [10–13]. Kirkley et al. [14] showed that valgus knee bracing can effectively reduce the pain symptom and joint mobility of KOA patients. However, Hunter et al. [15] found that valgus knee bracing can inhibit the pain symptom of KOA patients, but cannot elevate the functions of joint mobility. Therefore, it brings difficulty to accurately assess the effect of the valgus knee bracing in KOA. The previous systematic review reported by Brouwer also proposed this viewpoint [16]. It is demonstrated that conclusions of those published researches may be biased mainly because of the unmatured clinical randomized controlled trials (RCTs) they had performed. In addition, even if some of the studies had shown the positive outcomes of the valgus knee bracing, they are usually run in really short-term periods [17]. Thus, they cannot use to support the overall success of valgus knee bracing in different stages of treatment. Another study by Moyer et al. mainly focused on the biomechanical property of valgus knee bracing, but did not substantively propose the impact of valgus knee bracing in KOA [18]. Our project aims to systematically review and meta-analyze RCTs correlated the valgus knee bracing and clarify the impact of this therapy on pain release and functional improvement in KOA patients. Our findings will provide guidance and suggestions to the first-line clinical practices of physicians or surgeons.

## Methods

### Search strategy

PubMed, Embase, and Web of Science databases were searched by computer. The search period was from the inception of the database to May 2020. To supplement the literature, manually enter the references included in the study if necessary. The search terms include “Osteoarthritis,” “knee,” and “brace OR bracing OR Valgus brace OR Unloader brace.”

### Inclusion criteria

The inclusion criteria included the following: (1) RCTs of using valgus knee bracing to treat KOA; (2) in RCTs, the experimental group was given valgus knee bracing treatment, and the control group was given non-bracing and other conservative treatments (if there were more than one test group, these studies were also included); (3) the age, sex, race, nationality, and course of disease in the included studies are not limited; (4) RCTs have one or more of the following outcome indicators: WOMAC function score (Western Ontario and McMaster Universities Osteoarthritis Index, WOMAC), VAS pain score (visual analog scale, VAS), KOOS (Knee Injury and Osteoarthritis Outcome Score, KOOS), KOOS-ADL score (KOOS Activities of Daily Living, KOOS-ADL).

### Exclusion criteria

The exclusion criteria included the following: (1) non-clinical randomized controlled trials; (2) the conference included literature, reviews, and published the same research literature repeatedly in different languages; and (3) the outcome indicators data in RCTs cannot be used.

### Data extraction

Two researchers screened the literature separately, including the first step of screening after browsing the titles and abstracts of the literature, as well as reading the full text and determining whether it was finally included. After the data were included, the two researchers checked each other. If the opinions were not uniform, the third researcher should judge whether they were included. The relevant information extracted includes (1) basic information of the literature, including title, year, first author, and course of treatment, (2) incorporate the basic characteristics of RCTs and patient baseline data, (3) intervention measures of the two groups, (4) outcome indicators, and (5) key factors for evaluating the quality of literature. Microsoft Excel was used to record the relevant information. Besides, EndNote was used for document management.

### Quality assessment

The bias risk of the included studies was evaluated according to the Cochrane Handbook 5.0.1 RCT bias risk assessment tool. The quality of the literature is assessed by whether the included literature is a random method, whether there is allocation concealment, whether the blind method is used, whether the result data is complete, and whether the research results are selectively reported. Each result is divided into low risk, unclear, and high risk. The quality of the methodology was evaluated by two researchers, and if there were different opinions, the third researcher would participate in the discussion and resolved.

### Statistical analysis

Revman 5.3 software was used for data analysis. Heterogeneity analysis: The heterogeneity of the research results was tested by *χ*^2^. The size of heterogeneity is judged by *I*^2^. When *I*^2^ < 50%, a fixed-effect model should be used; when *I*^2^ > 50%, a subgroup analysis of the causes of heterogeneity may be performed; and when the difference between the two studies was not statistically significant, a random-effects model can be used for analysis. When *P* < 0.05, it means that the difference of the research results was statistically significant.

## Results

### Literature search

**Fig. 1:**
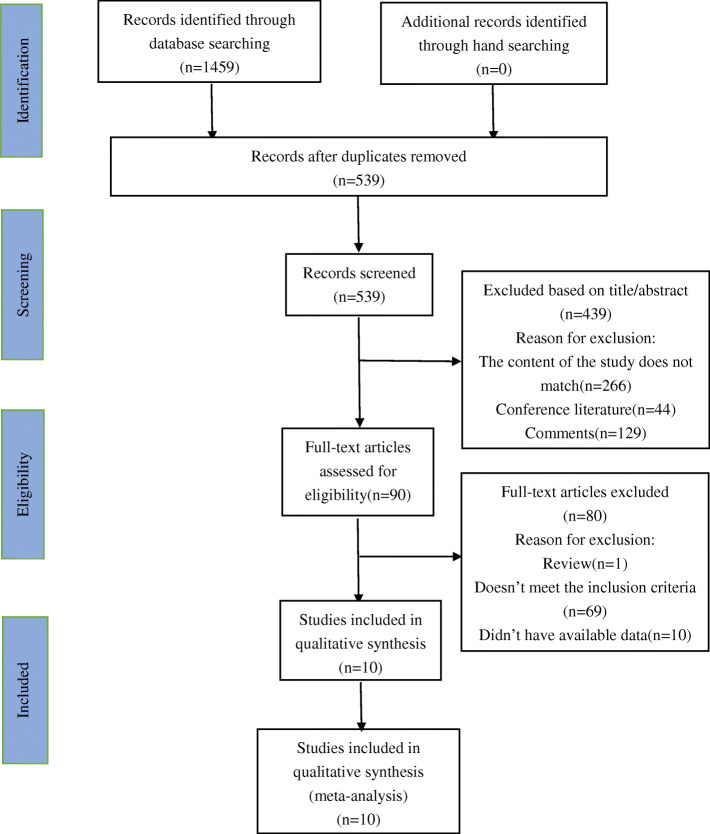
Flowchart of included studies

The literature search process was shown in Fig. 1. After preliminary search, a total of 1459 documents were identified in the three electronic databases. Among them, PubMed got 353 articles, Web of Science got 532 articles, and Embase got 574 articles. After excluding duplicate documents, there were 539 articles left. Subsequently, a total of 439 articles were excluded by reading the title and abstract, including 266 research contents that did not match, 129 review articles, and 44 conference documents. Of the remaining 90 articles, 80 articles were deleted after reading the full text, including 69 articles that did not meet the inclusion criteria, 10 articles that data were not available, and one review article. Finally, this study included 10 articles for meta-analysis totally.

### Study characteristics

A total of 739 patients were included in the 10 studies, including 412 patients in the experimental group and 414 patients in the control group. The minimum sample size was 10 and the maximum sample size was 86. Table 1 showed the detailed characteristics and main conclusions of all studies. Table 2 summarized the intervention measures for each study and the results of the outcome indicators.

**Table 1:** Characteristics of the studies in meta-analysis

Study	Year	Country/region	Journal	Study type	Level of evidence	Sample size (T/C)^a^	Gender (M/F)	Median age (T/C)^a^	BMI^a^ (T/C)^a^	Length of follow-up
Hunter et al. [15]	2012	Australia.	*Ann Rheum Dis*	RCT	I	29/27	30/50	63.0/60.0	32.7/34.7	30 weeks
van Raaij et al. [19]	2010	Netherlands	*Clin Orthop Relat Res*	RCT	I	46/45	46/45	54.9/54.4	29.0/29.4	26 weeks
Yu et al. [20]	2016	Australia.	*Int J Rheum Dis*.	RCT	II	86/68	54/100	67.7/67.0	30.7/33.2	52 weeks
Dammere et al. [21]	2018	Austria	*Knee Surg Sports Traumatol Arthrosc*	RCT	I	21/21	22/20	50.6/53.3	24.7/26.5	52 weeks
Thoumie et al. [22]	2018	France	*Sci Rep*.	RCT	I	32/35	23/44	64.8/66.6	29.2/28.1	6 weeks
Jones et al. [23]	2012	UK	*Gait Posture*	RCT	I	28/28	NA	NA	NA	2 weeks
Callaghan et al. [24]	2015	USA	*Ann Rheum Dis*	RCT	I	56/61	NA	NA	NA	6 weeks
Müller-Rath et al. [25]	2011	Germany	*Z Orthop Unfall*.	RCT	I	13/10	16/7	49.8/57.4	27/28	16 weeks
Niazi et al. [26]	2014	Pakistan	*Pak J Med Sci*	RCT	I	56/53	NA	NA	NA	26 weeks
Arazpour et al. [27]	2013	Iran	*Prosthet Orthot Int*	RCT	I	12/12	9/15	58.75/59.83	27.30/26.71	6 weeks

*C* control group, *BMI* body mass index, *NA* not applicable

^**a**^T experimental group

**Table 2:** Study interventions and outcome indicators

Lead author	Intervention (T)^**a**^	Intervention (C)^**a**^	Outcome indicators^**a**^
Hunter [15]	Valgus knee brace	A neutral knee brace (no valgus angulation)	②
van Raaij [19]	Valgus knee brace	Laterally wedged insole	①②
YU [20]	Valgus knee brace	No bracing	①③④
Dammere [21]	Valgus knee brace	A custom-made wedge insole	③④
Thoumie [22]	Valgus knee brace	No bracing	①
Jones [23]	Valgus knee brace	Laterally wedged insole	①②
Callaghan [24]	Valgus knee brace	No bracing	①④
Müller-Rath [25]	Valgus knee brace	An elastic knee bandage	①②
Niazi [26]	Valgus knee brace	Laterally wedged insole	①
Arazpour [27]	Valgus knee brace	Laterally wedged insole	①

*C* Control group

*T experimental group

^①^VAS (visual analog scale)

^②^WOMAC (Western Ontario and McMaster Universities Osteoarthritis Index)

^③^KOOS (Knee Injury and Osteoarthritis Outcome Score)

^④^KOOS-ADL (KOOS Activities of Daily Living)

### Quality assessment

**Fig. 2:**
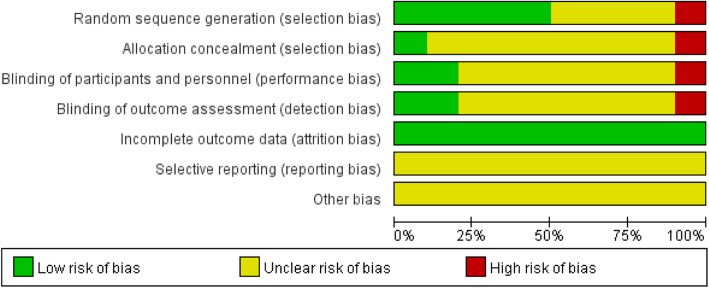
Individual risk bias included in the study

**Fig. 3:**
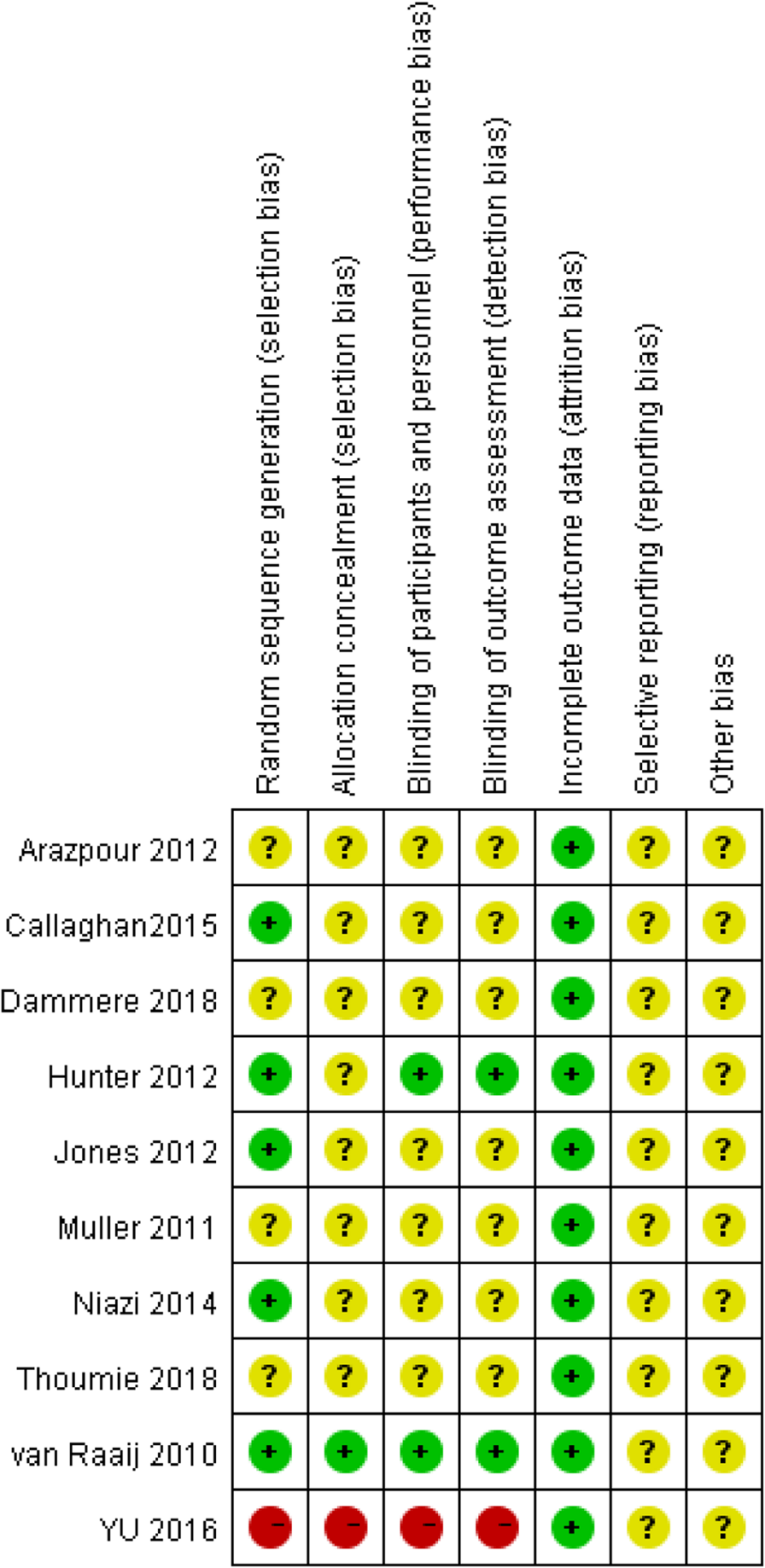
Risk of bias included in the study

Using the Cochrane Handbook 5.0.1 RCT bias risk assessment tool to evaluate the quality of 10 studies, the results are shown in Figs. 2 and 3. The figure showed a total of one high risk, and the rest were unclear or low risk. Overall, the quality of the literature was at the upper-middle level.

### Primary outcome measures

#### VAS pain score

**Fig. 4:**
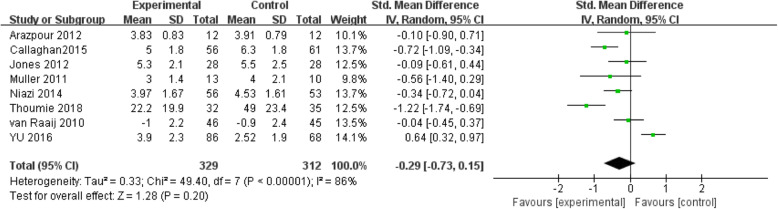
Meta-analysis of the VAS pain score

A total of eight studies reported the impact of valgus knee bracing on the VAS pain score of patients with KOA. The heterogeneity test results of the eight included studies were *I*^2^ = 88% > 50%, the random effect model was selected. The results showed that RR = − 0.29, 95% CI [− 0.73, 0.15], and the combined effect test *Z* = 1.28, *P* = 0.20 (Fig. 4), indicating that the experimental group compared with the control group had no statistically significant difference in improving knee VAS pain scores significance.

#### WOMAC function score

**Fig. 5:**
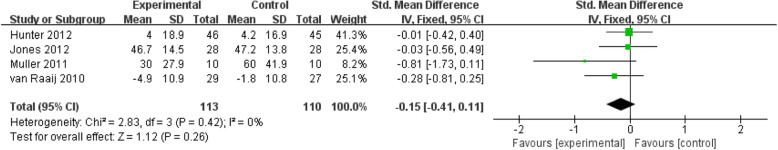
Meta-analysis of the WOMAC score

A total of four studies reported the effect of valgus knee bracing on the WOMAC function score of KOA patients. The heterogeneity test results of these four studies were *I*^2^ = 0 < 50%, so the fixed effect model was selected. As shown in Fig. 5, RR = − 0.15, 95% CI [− 0.41, 0.11]; the combined effect *Z* = 1.12, *P* = 0.26. This indicated that valgus knee bracing had no statistically significant difference in improving the knee WOMAC function score.

### Secondary outcome measures

#### KOOS score

**Fig. 6:**
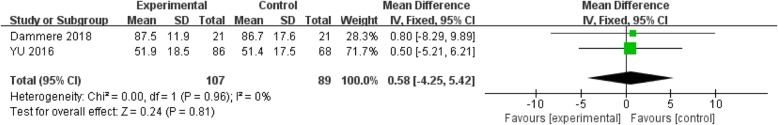
Meta-analysis of the KOOS score

A total of two studies reported the impact of valgus knee bracing on the KOOS score of KOA patients. The heterogeneity test results of these two studies were *I*^2^ = 0 < 50%, so the fixed effect model was chosen. The results can be obtained from Fig. 6: RR = 0.58, 95% CI [− 4.25, 5.42]; the combined effect *Z* = 0.24, *P* = 0.81. The results showed that the experimental group and the control group had no statistically significant difference in the KOOS score.

#### KOOS-ADL

**Fig. 7:**
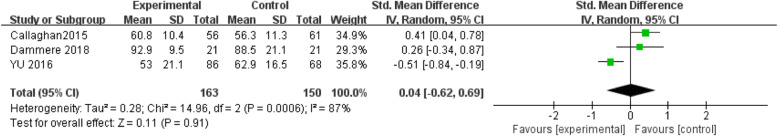
Meta-analysis of the KOOS-ADL score

A total of 3 studies reported the influence of valgus knee bracing on the KOOS-ADL score of KOA patients. The heterogeneity of the 3 studies was *I*^2^ = 87% > 50%, so the random effect model was elected. The specific results are shown in Fig. 7: RR = 0.04, 95% CI [− 0.62, 0.69]; the combined effect *Z* = 0.11, *P* = 0.91. The results showed that the difference between the KOOS-ADL scores of the experimental group and the control group was not statistically significant.

### Subgroup analysis

As can be seen from the above results, there were two outcome indicators with high heterogeneity, which were the VAS pain score and KOOS-ADL score. However, it is considered that the KOOS-ADL score was included in fewer studies, and the VAS score had the value of subgroup analysis. Through analysis of 8 studies that reported the impact of valgus knee bracing on VAS scores of KOA patients, it was found that the follow-up time of these 8 studies was very different, so we conducted a subgroup analysis of follow-up time. In the process of subgroup analysis, it was found that the calculation method of the VAS score in Thoumie’s [22] study is very different from other studies. After using or excluding this study, the heterogeneity had changed greatly. Therefore, the subgroup analysis excluded the Thoumie’s [22] study.

**Fig. 8:**
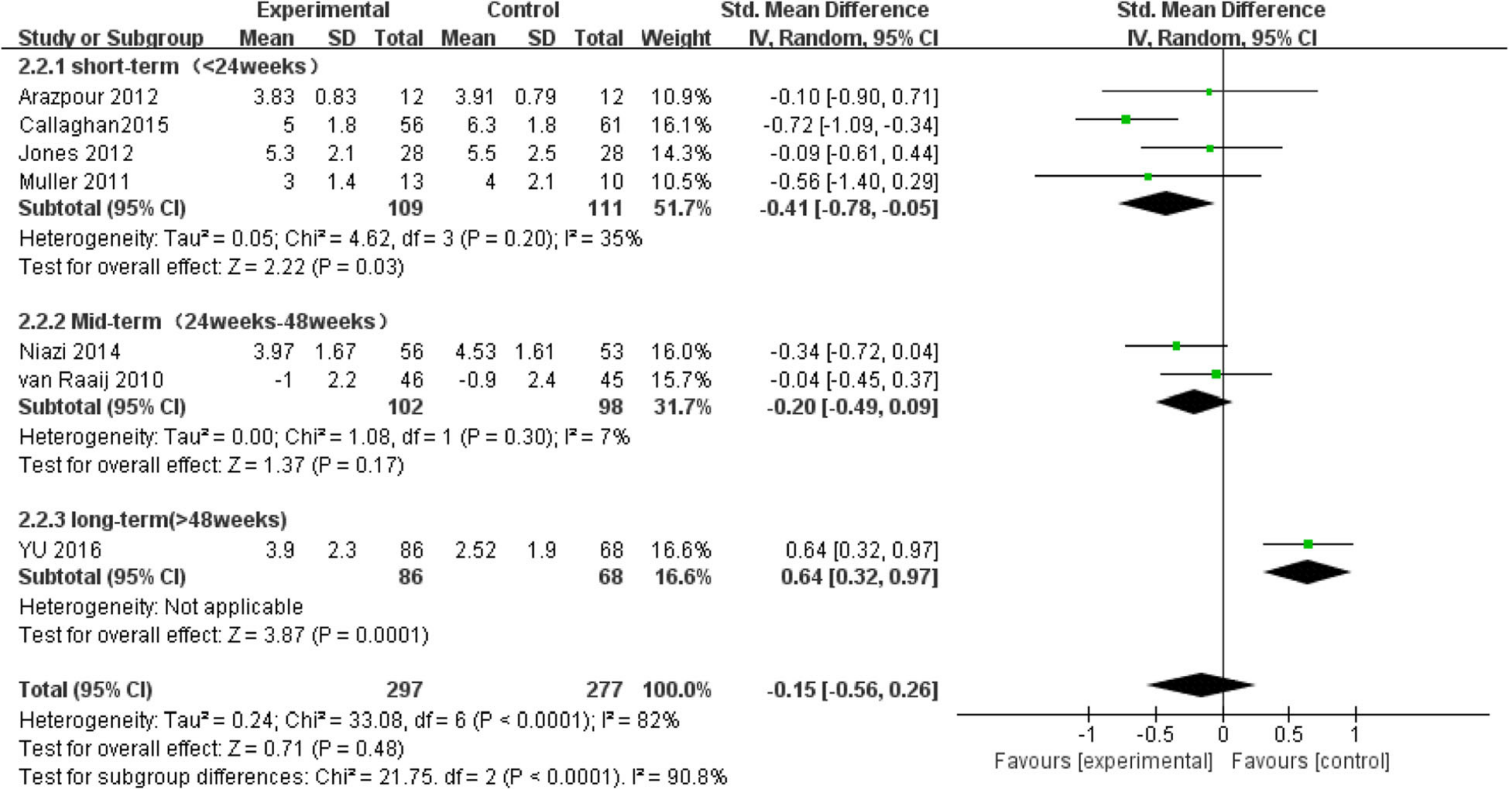
Subgroup analysis of the VAS pain score

The results of the subgroup analysis of the follow-up time are shown in Table 3 and Fig. 8. When the follow-up time was greater than 52 weeks, only one study was included, and there were no results of the heterogeneity; when the follow-up time was less than 24 weeks, the heterogeneity was *I*^2^ = 35%; when the follow-up time was between 24 and 48 weeks, the heterogeneity was *I*^2^ = 7%; and the results showed that the follow-up time was the source of the heterogeneity of the VAS pain score.

**Table 3:** Subgroup analysis of follow-up time of VAS score

Subgroup follow-up time	Results of subgroup analysis			
	Number of studies	MD* value (95%CI)	***P*** value	***I***^**2**^/%
a^**a**^ < 24 weeks	4 [21–24]	− 0.41 [− 0.78, − 0.05]	0.03	35%
24 weeks < a^**a**^ < 48 weeks	2 [17, 25]	− 0.20 [− 0.49, 0.09]	0.17	7%
a^**a**^ > 48 weeks	1 [18]	0.64 [0.32, 0.97]	0.0001	–

*MD* mean difference

^**a**^a follow-up time

### Adverse reactions

None of the 10 articles included in this study reported adverse reactions.

## Discussion

Currently, multiple conservative therapies for knee osteoarthritis (KOA) are applied in clinical practices, such as weight control, exercise, physical therapy (including acupuncture, laser therapy, and electromagnetic therapy), oral analgesics, and joint cavity injection drugs [28, 29]. Emerging studies paid attention to the changes of force lines in the knee joint. An unbalance of weight-bearing will somehow change the knee force lines and cause lower limb abnormality, leading to the progress of KOA [10, 11]. Theoretically, the valgus knee bracing can optimize the direction of force line in the knee joint, further reducing pain symptoms and improving joint function [12, 13]. However, the clinical efficacy of valgus knee bracing in the treatment of KOA is not clear. Surprisingly, different studies show opposite outcomes which seriously troubles the selections of physicians in clinic [15–27, 30–38]. Therefore, a meta-analysis is urgent to be performed in order to clarify the efficacy of valgus knee bracing.

Our findings demonstrated that valgus knee bracing did not improve the VAS pain score (*P* = 0.29), WOMAC function score (*P* = 0.26), KOOS (*P* = 0.81), and KOOS-ADL (*P* = 0.91). It is indicated that valgus knee bracing cannot improve the pain, activity function, and quality of the daily life of KOA patients. Due to the high heterogeneity of the VAS pain score, we conducted a subgroup analysis of the follow-up time. The results showed that valgus knee bracing enable to improve the VAS pain score of KOA patients if the follow-up period was less than 24 weeks, (*P* = 0.03). In contrast, a negative results were obtained if the follow-up time was between 24 and 48 weeks (*P* = 0.17), indicating that valgus knee bracing could not improve the pain symptoms of KOA patients in a long term. When the follow-up time was greater than 48 weeks, the result was *P* = 0.0001. But VAS pain score of the experimental group was found to be greater than that of the control group, and it is still shown that valgus knee bracing cannot improve or even increase the pain symptoms of KOA patients.

Duivenvoorden et al. [36] further confirmed the conclusions of this study. They conducted a secondary analysis of randomized controlled trial data in 80 patients with KOA and found no biomechanical evidence to support the use of valgus knee bracing. Even though the meta-analysis performed by Moyer et al. [17] showed that valgus knee bracing can improve joint activities in patients with KOA, however, references enrolled in this study were mainly short-term research. Specifically, if excluding one study with a 52-week follow-up, the follow-up time of the remaining studies was less than 24 weeks. Therefore, it still remained undetermined about the long-term efficacy of valgus knee bracing. Another study by Moyer et al. [18] focused on the biomechanical property of valgus knee bracing but did not substantively verify the effect of valgus knee bracing on pain symptoms and functional activity of patients with KOA. Furthermore, only three of the thirty studies included in the study of Moyer et al. [18] are in long-term follow-up. The meta-analysis by Cudejko et al. [39] also focused on short-term indicators only. Our study includes only ten studies with 739 patients. Nevertheless, six of them are followed up for more than 24 weeks, which provides stronger evidence to the long-term results.

It is surprising that the outcome of our meta-analysis is different from others. We concluded the following reasons to explain low efficiency of valgus knee braces in long-term observation. Firstly, the compliance of patients is low in valgus knee bracing treatment. Few patients used valgus knee bracing for more than 4 h a day. Therefore, the effectiveness of valgus knee bracing may be underestimated in the long-term period [40]. Secondly, studies have suggested that, if in valgus knee bracing, it will make the patient believe that the knee joint on the brace side is injured and subconsciously use the limb on the other side [41]. The movement of the knee joint on the brace side will be reduced, which will negatively affect the improvement of the stiffness of the knee joint as well as the recovery of the joint function, and further affect the improvement of the pain symptoms. Thirdly, the valgus knee bracing mainly provides valgus moment of force for the knee joint. Studies have shown that valgus knee bracing reduced the external knee adduction, but not being beneficial to the external flexion moment and free moment, which are critical factors in the development of KOA [42]. Fourthly, Creep [43] indicated that knee deformation will increase under constant weight-bearing load. The valgus knee bracing will produce creep during long-term use. Therefore, the reason why the long-term treatment effect of the valgus knee braces is not ideal may be related to the creep of the material. Therefore, only the above problems can be solved, and valgus knee bracing can truly contribute to the KOA treatment.

The limitations of this study are as follows: (1) due to the small amounts of RCTs enrolled, publication bias is unavoidable in our project, and (2) the valgus knee braces used in the testing group of RCTs are created in different shapes and construction by different manufacturers. The intervention time of valgus knee bracing in the control group are usually not the same. These above factors lead to clinical heterogeneity; (3) it is impossible to enroll all relevant studies that have been reported whereas our search filed covered PubMed, Embase, and Web of Science databases; (4) the RCTs included in this study did not provide complete outcome indicator data, which led us to use statistical methods to determine the outcome indicators data based on the information provided. But after we excluded this study, the results were not affected.

## Conclusion

Taken together, our current evidence shows that valgus knee bracing can only improve knee joint activities and relieve pain feelings of KOA patients in a short-term therapy, but showing no contribution to the long-term outcomes. More high-quality clinical RCTs with longer follow-up time are needed to further verify our conclusions.

## Data Availability

The datasets used and/or analyzed during the current study are not publicly available due to feasibility but are available from the corresponding author on reasonable request.

## Abbreviations

OA: Osteoarthritis
KOA: Knee osteoarthritis
TKR: Total knee replacement
WOMAC: Western Ontario and McMaster Universities Osteoarthritis Index
VAS: Visual analog scale
KOOS: Knee Injury and Osteoarthritis Outcome Score
KOOS-ADL: KOOS Activities of Daily Living
RCTs: Randomized controlled trials
